# Large Language Models in Genomics—A Perspective on Personalized Medicine

**DOI:** 10.3390/bioengineering12050440

**Published:** 2025-04-23

**Authors:** Shahid Ali, Yazdan Ahmad Qadri, Khurshid Ahmad, Zhizhe Lin, Man-Fai Leung, Sung Won Kim, Athanasios V. Vasilakos, Teng Zhou

**Affiliations:** 1 School of Cyberspace Security, Hainan University, Haikou 570228, China; ali.ali.md111@gmail.com (S.A.); linzhizhe@hainanu.edu.cn (Z.L.); 2 School of Computer Science and Engineering, Yeungnam University, 280, Daehak-ro, Gyeongsan-si 38541, Gyeongsangbuk-do, Republic of Korea; yazdan@yu.ac.kr (Y.A.Q.); swon@yu.ac.kr (S.W.K.); 3 Department of Health Informatics, College of Applied Medical Sciences, Qassim University, Buraydah 51452, Saudi Arabia; k.ahmad@qu.edu.sa; 4 School of Computing and Information Science, Anglia Ruskin University, Cambridge CB1 1PT, UK; man-fai.leung@aru.ac.uk; 5 Department of Information and Communication Technology, University of Agder, 4879 Grimstad, Norway

**Keywords:** large language models (LLMs), artificial intelligence (AI), genomic data, precision medicine

## Abstract

Integrating artificial intelligence (AI), particularly large language models (LLMs), into the healthcare industry is revolutionizing the field of medicine. LLMs possess the capability to analyze the scientific literature and genomic data by comprehending and producing human-like text. This enhances the accuracy, precision, and efficiency of extensive genomic analyses through contextualization. LLMs have made significant advancements in their ability to understand complex genetic terminology and accurately predict medical outcomes. These capabilities allow for a more thorough understanding of genetic influences on health issues and the creation of more effective therapies. This review emphasizes LLMs’ significant impact on healthcare, evaluates their triumphs and limitations in genomic data processing, and makes recommendations for addressing these limitations in order to enhance the healthcare system. It explores the latest advancements in LLMs for genomic analysis, focusing on enhancing disease diagnosis and treatment accuracy by taking into account an individual’s genetic composition. It also anticipates a future in which AI-driven genomic analysis is commonplace in clinical practice, suggesting potential research areas. To effectively leverage LLMs’ potential in personalized medicine, it is vital to actively support innovation across multiple sectors, ensuring that AI developments directly contribute to healthcare solutions tailored to individual patients.

## 1. Introduction

Personalized or precision medicine utilizes genomic, environmental, and lifestyle data to inform healthcare decisions, marking a paradigm shift in medical practice [1]. This strategy diverges from conventional methods by employing personalized therapies tailored to the unique characteristics and needs of each patient [2]. Traditional therapeutic approaches prioritize treating patients over individual characteristics such as genetics, health status, physical condition, age, and gender, necessitating personalized medicine. As a result, the efficacy of drugs and therapies varies significantly between patients, ranging from highly effective to completely ineffective [3]. Medical research focuses on developing individualized diagnostic procedures and innovative medications tailored to each patient’s specific needs. Advances in high-resolution analytics, pharmacogenomics, biotechnology, chemistry, and cell and molecular biology are critical for developing effective drugs and gaining a deeper understanding of genetic and biological processes. Disease-specific biomarkers provide precise information about the nature, molecular origin, and progression of diseases, allowing for the successful implementation of personalized therapeutic approaches. Diseases can be classified according to their type, molecular cause, and stage, allowing for more effective implementation of personalized therapeutic strategies [4]. Personalized medicine combines interdisciplinary medical professionals and integrated technologies to improve disease understanding and preventive measures. To achieve the best results, personalized medicine tailors treatments to an individual’s unique genetic makeup, environment, and lifestyle. This approach relies heavily on genomic data analysis, which provides in-depth insights into genetic tendencies, disease mechanisms, and treatment responses [5].

**Figure 1:**
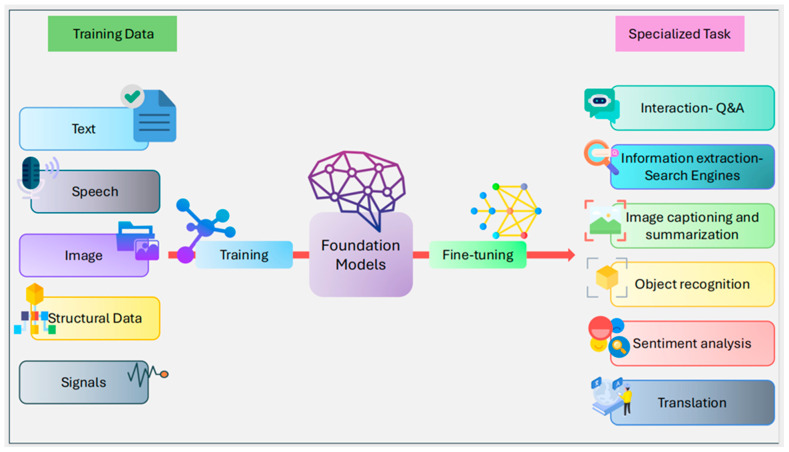
Foundation models. FMs are highly versatile and can be fine-tuned to adapt to perform specialized tasks (translation, object recognition, sentiment analysis, etc.).

The ubiquity of artificial intelligence (AI) is undoubtedly a result of the fast-paced evolution of hardware and software. Large language models (LLMs) represent the next step in the evolution of AI, which has significantly altered how humans interact with technology. Search engines, writing tools, image and video generation, and software development are among the key areas where LLMs have triggered a paradigm shift [6]. LLMs can identify natural language input and generate output using knowledge based on billions of resources available online and application-specific databases. LLMs can recognize the context of the input prompt and can, therefore, generate a relevant and highly accurate output [7]. Foundational models (FMs) are highly versatile learning models trained on a relatively small dataset and are capable of performing a wide variety of tasks. However, LLMs require a large corpus of domain-specific data to perform specific tasks based on natural language prompts. An LLM can be considered an FM that is trained on a very large, specialized dataset to perform a dedicated task, such as language translation or text generation. FMs are essentially deep learning (DL) neural network (NN)-based models that are highly versatile and adaptive. FMs utilize unlabeled data for self-supervised learning and minimal fine-tuning to specialize in a wide range of applications, as illustrated in Figure 1. Consequently, they form the foundation for many AI applications, including text prediction, image generation, and biomedical image analysis [8,9]. FMs are built using powerful NNs, including transformers, generative adversarial networks (GANs), diffusion models, and variational autoencoders.

LLMs are based on transformers that were introduced in 2017 in a seminal work published by Google called “Attention Is All You Need” [10]. Transformers are NNs that are capable of understanding the context of each element of an input by identifying its relationship with other input elements. Transformers create an algebraic map to define a relationship between each input element by utilizing positional encoders [11]. Transformers convert the input sequence into tokens, which are an algebraic representation of each input element. The model is trained on a large volume of training data, which enables the model to understand the relationship between each token and its neighbors [12]. Each token can be processed in parallel, which accelerates the training process. LLMs can accurately translate text, generate highly detailed and accurate text data, interpret and summarize text, and even perform peer reviews [6]. LLMs are also successfully utilized for code and image generation [13,14]. LLMs can revolutionize healthcare services and research through the use of vast medical records and datasets to train for disease diagnosis, protein discovery, and accelerate drug development [15].

### 1.1. Motivation

In 2021, the United States Food and Drug Administration (FDA) evaluated over one hundred applications containing AI components, indicating a significant shift toward incorporating AI in healthcare submissions. AI rapidly improves the accuracy and refinement of disease-specific advanced therapy medicinal products (ATMPs) [16]. LLMs can assist medical professionals in the clinical decision process, leveraging their capability to correlate patient conditions with the medical literature and derive conclusions. In addition, medical professionals can rely on the LLMs to gather and process relevant information to support their clinical decisions. Several clinical decision-support LLMs have been developed to understand clinical terminology [17]. LLMs can support biomedical research and drug development by analyzing the massive tranche of academic literature available on this topic [18,19].

### 1.2. Contributions

This review aims to highlight the transformative potential of LLMs in personalized medicine, particularly through the analysis of genomic data. Recent advances in AI, particularly the development and refinement of LLMs, have paved the way for more sophisticated analyses of large genomic datasets. These models can interpret the complex language of genetics, predict outcomes, and provide insights at unprecedented scale and accuracy. By leveraging LLM capabilities, researchers can perform more nuanced, faster, and potentially less expensive genomic analyses. This enables a more tailored approach to medicine, considering individual genetic backgrounds and increasing the efficacy of treatments and interventions. The contributions of this survey can be summarized as follows:

1. Present a primer to LLMs and their architecture.
2. Understand the LLMs in genomics from the perspective of personalized medicine.
3. Identify the limitations of LLMs and possible future research directions in this domain.

### 1.3. Related Work

The existing surveys investigating the efficacy of LLMs in personalized medicine, especially in the context of genomics, are rudimentary. These works focus on the applications, operational terminology, and tutorials of LLMs in healthcare. The scope of these surveys is usually limited to clinical decision support systems, clinical information search and delivery, and biomedical research and education. To the best of our knowledge, this work is the first comprehensive survey to focus on personalized medicine, with an emphasis on the genomic aspect of precision medicine. The survey [20] introduces LLMs and their role in biomedical applications. This work provides a broader examination of the fundamental architecture of LLMs, their applications, and utilization strategies to enhance model performance, ultimately identifying the challenges associated with LLMs. The authors of [21] use a similar approach and delve deeply into the various applications of LLMs, discussing personalized patient management in a limited capacity. The study [22] offers a technical insight into the role of LLMs in medicine and discusses the technical challenges associated with these models. The term Med-LLMs was coined in [23] to refer to LLMs that are designed for medical tasks. These tasks include clinical decision support, medical literature inference, report generation, and medical education. This work does not cover personalized medicine in detail. Another general scope study is presented in [24], which covers the latest advancements in LLMs and their contribution to advancing healthcare. Their discussion also provides insight into the ethical and social implications of LLMs, particularly in light of the privacy concerns surrounding the sharing of sensitive medical data. The surveys [20,21,22,23,24] focus on the natural language processing (NLP) capabilities of LLMs. A brief survey undertakes a study of transformer-based models that are used to accelerate the speed of drug development [25]. The process of developing new pharmaceutical drugs requires an intensive study of chemical interactions, which help establish the efficacy and safety of a drug before the clinical trial starts. The LLMs accelerate this process by identifying adverse impacts based on their ability to analyze the vast scientific literature and streamline the drug development process. A similar but comprehensive study in [26] covers the drug discovery process and the role of LLMs in the process. The development of personalized medications can benefit from the contextual awareness that LLMs provide. The interaction of proteins determines the efficacy of a drug and possible adverse reactions that might result due to genetic predispositions. Therefore, the capabilities of LLMs can be used for understanding protein interactions and designing proteins to enhance personalized treatments. Surveys [27,28] provide a deep insight into these interactions and their potential role in developing personalized treatment plans.

Section 2 introduces LLMs and their role in healthcare, especially in medical research. It delves into the building blocks of LLMs and different model architectures. Section 3 introduces the key concepts of precision medicine, discussing the various aspects of personalized healthcare and its key enabling techniques. We discuss the role of LLMs in advancing precision medicine in personalized healthcare systems and the underlying medical research in Section 4. The limitations and potential research direction are laid out in Section 5. Section 6 concludes this discussion.

## 2. Large Language Models: An Introduction

**Figure 2:**
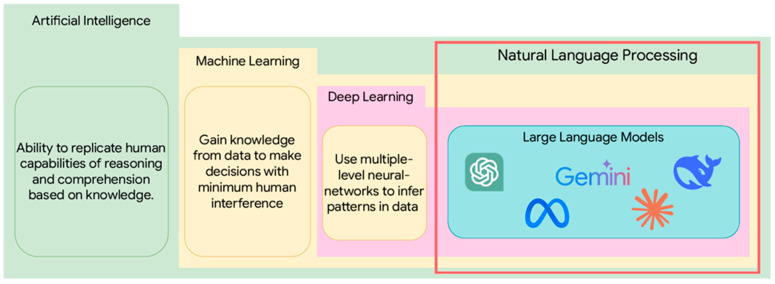
The relationship between AI, ML, DL, and LLM, illustrating how LLMs merge NLP capabilities with the advanced learning and cognitive functions provided by AI.

ChatGPT, from OpenAI (San Francisco, CA, USA), is an LLM that has changed the landscape of the use of AI in the consumer space and the research community. At least four research articles co-authored by ChatGPT have been accepted during peer review [29]. ChatGPT has evolved since 2020, with ChatGPT-4 passing the Turing Test [30]. Google and Meta AI also released their latest LLMs, Gemini and Llama 3. These exponentially large models are available for various applications, including research and development. LLMs are based on the transformer architecture, essentially deep NNs trained on a large corpus of data. Therefore, LLMs are a class of DL models, a machine learning (ML) subgroup. The relationship between AI, ML, DL, and LLMs can be illustrated in Figure 2.

**Figure 3:**
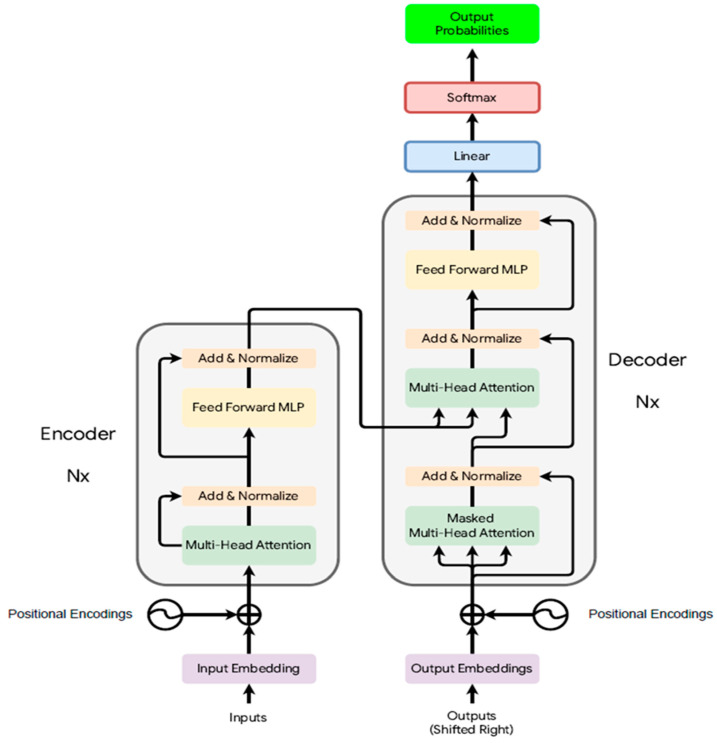
The architecture of a transformer introduced in [10].

Transformers are essentially DL models that are trained using unsupervised and reinforcement learning (RL) combined with human feedback. A transformer is composed of several layers of NNs, as illustrated in Figure 3. The transformer comprises an encoder and a decoder, each containing identical stacked layers. Both include a multi-head self-attention mechanism and a position-wise fully connected feed-forward network. The self-attention mechanism of the encoder allows the model to weigh the different tokens in an input sequence with varying importance by projecting the input embeddings into query, key, and value spaces [31]. The attention scores are computed as a dot product between the query and key, determining a weighted sum of the values. There are multiple attention blocks known as attention heads. This allows the different attention heads to capture various dependencies in the sequence in parallel. The decoder resembles the structure of the encoder with an additional mechanism of encoder–decoder attention, which allows for coherent and contextually relevant output sequences by attending to the entirety of the encoded input. The decoder has an additional masked multi-head attention layer, which ensures that the output sequence is generated based only on the previously generated tokens and not future ones [32]. To address the above-mentioned inadequacy in intrinsic position awareness, positional encodings augment the input embeddings to retain sequential information, differentiating transformers from previous attention mechanisms. Each layer’s feed-forward network elevates the representations using non-linear transformations. Stability and better gradient flow during training are enhanced by using residual connections and performing layer normalization. This model serves as a basis for many state-of-the-art models, such as Bidirectional Encoder Representations from Transformers (BERTs), Bidirectional and Auto-Regressive Transformers (BARTs), and Generative Pre-trained Transformers (GPTs) (Figure 3).

Transformers can be broadly classified into three categories based on their architectural design [33] as follows: 1. encoder-only models, 2. decoder-only models, and 3. hybrid models.

### 2.1. Encoder-Only Models

These classes of LLMs are also known as BERT-style models. These models are based on bidirectional pre-training, which means that the context of the input is understood from both directions, from the left and right of each input token. These are primarily encoder-only models, which can predict masked words based on the context provided by the surrounding words, thereby earning the moniker “masked language modeling”. These are non-causal models that do not regard the causality of the language. All the tokens are visible to each other during the attention process, disregarding the causal relationship between the individual tokens. BERT and the Robustly Optimized BERT Pretraining Approach (RoBERTa), developed by Google and Meta AI, fall into this category [34,35].

### 2.2. Decoder-Only Models

LLMs are fundamentally designed to be task-agnostic, but they can be trained on specific datasets to perform specialized tasks in a process termed fine-tuning [36]. However, scaling up the language models can significantly improve performance in both zero-shot and one-shot learning [37]. Zero-shot learning refers to the model’s ability to understand a data sample without prior knowledge of the data class. In contrast, one-shot learning means the model has been trained once on a data class before a sample is taken as the input. Unlike encoder-only models, these models are causal models that consider the preceding words to predict the next word in a generated output sentence. Therefore, the model only considers the tokens preceding the current token, ensuring the causality in the generated output. Generative pre-trained transformers (GPTs) fall into this category; therefore, this category is also referred to as GPT-style models. The GPT-4 architecture is an autoregressive language model that falls into this category [37].

### 2.3. Hybrid Models

Hybrid models combine the best of both worlds, from encoder and decoder models, and introduce pre-training. The UNILMv2 model is introduced in [38], which belongs to the pseudo-masked language model (PMLM) class. This approach combines the attention procedure of the encoders in the encoder–decoder models in the first part, and the second part mirrors the decoder functionalities. BART [39] is another example of a hybrid model that is suitable for text comprehension and generation.

Researchers continue to explore and refine these architectures, pushing the boundaries of what LLMs can achieve for different applications [40]. An LLM architecture can be selected for a specific task based on several considerations. For bidirectional contextualization, encoder-only models like BERT and RoBERTa may be more suitable. For generation tasks, such as translation or summarization, decoder-only or encoder–decoder models, like the GPT-style models or BART, may be suitable candidates. Table 1 presents LLMs and their underlying architectures. It also highlights their creators and their applications.

**Figure 4:**
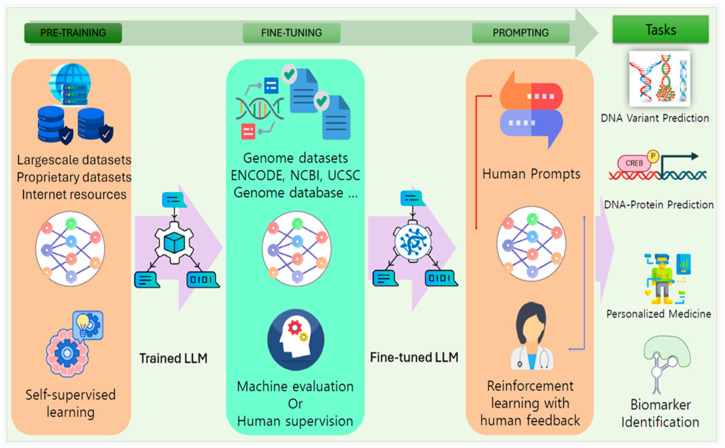
The process to train an LLM for biological applications.

The integration of LLMs in computational biology and bioinformatics has accelerated the process of drug discovery and protein identification. LLMs initially designed for NLP tasks have shown remarkable adaptability and effectiveness in understanding and generating biological sequences due to their versatility and ability regarding zero-shot and one-shot learning [47]. LLMs are fine-tuned by training the FMs on application-specific datasets for genome study, protein identification, and disease prediction, as illustrated in Figure 4.

LLMs have been adapted to assist in predicting molecular properties, identifying potential drug candidates, and optimizing drug design, which is usually a resource and time-intensive process. ChatMol is a novel approach to molecular discovery that combines natural language capabilities with drug design and molecular research [48]. ChemBERTa leverages a transformer model called RoBERTa [35] to encode chemical information directly from a Simplified Molecular Input Line Entry System (SMILES) dataset. This model utilizes self-supervised learning to understand chemical properties and interactions, facilitating drug discovery processes such as lead identification and optimization [49]. NVIDIA offers a generative AI platform to accelerate drug development, utilizing its proprietary NVIDIA BioNeMo platform. This platform enables researchers to run multiple FMs, including ESM-1, OpenFold [50], MegaMolBART, and ProtT5.

Protein identification involves predicting protein structures, functions, and interactions, which are crucial for understanding biological processes and developing effective therapeutic strategies. DL models are available for predicting protein structures and their molecular interactions. AlphaFold2, developed by Google’s DeepMind, is a breakthrough DL model that accurately predicts protein structures. Its creators were awarded the 2024 Nobel Prize in chemistry due to its potential to transform scientific discovery. It uses self-attention to infer the three-dimensional structure of proteins from their amino acid sequences, aiding in the understanding of protein function and interaction [51]. AlphaFold3, released in 2024, demonstrates high prediction accuracy in detecting almost all the protein types in the Protein Data Bank (PDB) along with a broader range of biomolecular complexes, including ligands, metals, and modified residues [52]. It uses a generative diffusion-based approach compared to an evoformer used in AlphaFold2. OpenFold released a reimplementation of AlphaFold2 designed to be fast, memory-efficient, and trainable from scratch, providing an open framework for protein structure prediction [50], which matches the accuracy of AlphaFold2. While AlphaFold is a complex DL model, OpenFold released an LLM called SoloSeq, which is 10× faster but delivers comparable performance to AlphaFold2. GenoML is a Python package (v1.0.1) that is used in automating genomics research [53].

LLMs have been adapted to interpret genomic data, identify variants, and predict their effects. BERT-genome adapts the BERT architecture to genomic sequences. The BERT architecture is designed to pre-train deep bidirectional representations from an unlabeled text by conditioning on both the left and right context in all layers simultaneously. An additional output layer can be used to fine-tune the BERT model, resulting in novel models for diverse tasks without requiring substantial modifications to the task-specific architecture. ProteinBERT applies a transformer architecture to protein sequences. This model learns contextualized representations of protein sequences which are beneficial for various tasks, including protein classification, function prediction, and interaction analysis [54]. The authors of [55] compared the performance of auto-regressive and auto-encoder models for protein identification, yielding exceptionally accurate results. DNABERT employs transformer models to capture patterns in DNA sequences. This model enhances the ability to identify genomic variants and predict their potential impacts on gene function and disease [56]. These models are fine-tuned for medical applications for diagnosis and disease prediction using electronic health records (EHRs) [57,58,59,60]. Table 2 summarizes the LLM models that are specialized for biological research and medicine, while Table 3 lists the common databases used to access training datasets for these models.

## 3. Personalized Medicine

Precision medicine, also known as personalized medicine, is a medical approach that advocates for customizing healthcare by tailoring medical decisions, treatments, practices, or interventions to each individual patient [66]. This strategy utilizes diagnostic tests to determine the most suitable and effective treatment plans, informed by a patient’s genetic makeup or other molecular or cellular studies. The concept of customized medicine is based on the unique response of each patient to the treatment plan. However, the absence of technological breakthroughs has hindered the comprehension and implementation of personalized medical interventions [67].

The completion of the Human Genome Project (HGP) in 2003 marked a significant milestone in the history of precision medicine. The HGP provided the first comprehensive map of all human genes, enabling researchers to understand the genetic basis of many diseases and conditions [68,69]. This study paved the way for developing tools and procedures to analyze an individual’s genetic information, making personalized medicine a more viable notion. Following the HGP, the field of genomics experienced significant growth. Advances in sequencing technology, such as next-generation sequencing (NGS) [70], have reduced the cost and time necessary to sequence a genome, making it suitable for widespread clinical use. The current push to integrate genomic data with clinical practice yields more precise diagnoses and targeted therapies [71].

Precision medicine aims to enhance treatment efficacy by tailoring medical interventions to each patient’s unique genetic traits. Understanding the genetic and molecular underpinnings of an illness allows clinicians to choose treatments that are more likely to benefit a specific patient [72]. This technique reduces the trial-and-error aspect of traditional medicine by providing treatments based on population averages rather than individual needs [73] and aims to minimize side effects by tailoring therapies to the patient’s genetic composition. Precision medicine has the potential to be cost-effective in the long run by providing more effective treatments and reducing the incidence of adverse effects. This can reduce the healthcare costs associated with prolonged therapies, hospitalizations, and managing side effects [74].

### 3.1. Precision Medicine and Genomic Analysis

Precision medicine consists of multiple interconnected components that deliver a personalized approach to patient care. These include genomic data analysis, biomarker discovery, pharmacogenomics, and clinical applications [4].

#### 3.1.1. Genomic Data Analysis

Genomic data analysis examines an individual’s genetic material to identify mutations and variations that may influence disease risk, progression, and response to treatment. This analysis reveals the extent of genetic variability in the human population and its implications for health and disease. Single-nucleotide polymorphisms (SNPs), insertions, deletions, and copy number variations (CNVs) are among the types of genetic variations that can impact an individual [75,76]. Several technologies and methodologies are employed in genomic data analysis, including whole-genome sequencing (WGS) [77], whole-exome sequencing (WES) [78], and targeted gene panels [79]. These technologies have changed the capacity to analyze the genome swiftly and cost-effectively, making them useful in research and therapeutic contexts [71].

#### 3.1.2. Biomarker Identification

Biomarkers play a critical role in precision medicine by providing information regarding the type, etiology, and stage of a disease, thereby informing personalized therapeutic approaches. Measurable indicators in blood, urine, tissues, or imaging scans encompass nucleic acids, deoxyribonucleic acid (DNA) and ribonucleic acid (RNA), proteins, lipids, cells, and imaging characteristics. These are essential for disease diagnosis, prognosis, and treatment selection, enhancing the precision and effectiveness of medical interventions [80,81]. Identifying biomarkers is an important part of precision medicine as it provides insights into disease causes and aids in developing targeted treatments. For example, the discovery of the HER2 protein as a biomarker in breast cancer has led to the development of targeted medicines such as trastuzumab [82,83].

#### 3.1.3. Pharmacogenomics

Pharmacogenomics is an essential aspect of precision medicine as it investigates how genetic differences influence individual responses to drugs. Pharmacogenomics enables personalized treatment by identifying genetic markers that influence drug metabolism, efficacy, and safety [84]. Pharmacogenomics examines how genes affect an individual’s response to medications. This discipline aims to enhance medication therapy by tailoring it to the patient’s genetic composition, thereby boosting efficacy while minimizing side effects. For instance, changes in the CYP2C9 and VKORC1 genes [85] impact the metabolism of warfarin [86], a routinely used anticoagulant, necessitating individualized dosing regimens to prevent bleeding problems.

### 3.2. Key Enablers of Personalized Medicine

Over the past decade, genomic data processing has made significant progress, thanks to technological advancements. Genomic data analysis is crucial in predicting and preventing diseases. Individuals’ risk profiles can be categorized based on genetic risk factors for specific diseases, allowing for focused preventive treatments [87]. Individuals with a high hereditary risk for cardiovascular illnesses can be evaluated more regularly and offered specific lifestyle and pharmaceutical measures to minimize their risk [88]. Using genomic data in customized treatment presents some ethical, legal, and social concerns [89]. Privacy and confidentiality of genetic information, informed consent, the possibility of genetic discrimination, and fair access to genomic-based healthcare are some of the issues that must be addressed to ensure the responsible use of genomic data. It is critical to develop ethical norms and regulatory frameworks to address these difficulties and encourage the equitable use of precision medicine [90]. The following discussion delves into the key innovations that are driving personalized medicine research.

#### 3.2.1. Next-Generation Sequencing

NGS remains at the forefront of genomic data processing, enabling high-throughput sequencing of DNA and RNA with remarkable speed and accuracy. NGS technologies have dramatically lowered the cost of sequencing, making it accessible for a wide range of applications, including research and clinical diagnostics [91]. Building on NGS’s success, third-generation sequencing technologies [92], such as Pacific Bioscience’s Single Molecule Real-Time (SMRT) sequencing and Oxford Nanopore Technologies’ nanopore sequencing, provide longer read lengths and the ability to sequence single molecules of DNA or RNA in real-time. These methods provide more comprehensive insights into complex genomic regions, structural variations, and epigenetic alterations [93].

#### 3.2.2. Single-Cell Genomics

Single-cell genomics is a novel discipline that allows for the investigation of genetic and transcriptional variability at the individual cell level. Unlike typical bulk sequencing, which averages signals across millions of cells, single-cell genomics captures the heterogeneity of cellular populations, exposing unique cell types, states, and lineages within a given sample. Single-cell RNA sequencing (scRNA-seq) is an essential technology in this field. It profiles the transcriptomes of individual cells by isolating single cells, reverse transcribing their RNA into cDNA, amplifying the cDNA, and then sequencing it [94]. This approach has transformed our knowledge of cellular diversity, developmental biology, and disease pathways. In developmental biology, scRNA-seq maps gene expression profiles at various stages of development, identifying new cell types and reconstructing developmental trajectories [95]. In cancer research, single-cell genomics unravels tumor heterogeneity by analyzing the genetic and transcriptomic profiles of individual cancer cells, identifying subpopulations with distinct mutations and transcriptional programs crucial for understanding tumor evolution, metastasis, and drug resistance [96]. DL has demonstrated potential in enhancing single-cell omics by surpassing conventional data preprocessing and analysis models, although its complete capabilities in tackling significant challenges remain unexploited [97].

#### 3.2.3. Gene Editing Technology

Gene editing technologies, primarily Clustered Regularly Interspaced Short Palindromic Repeats (CRISPRs) technology, have revolutionized the field of genetics by providing a precise and efficient method for modifying DNA sequences. CRISPR Cas9, the most widely used system, utilizes a guide RNA to target specific DNA sequences, where the Cas9 enzyme creates double-strand breaks. These breaks are then repaired by the cell’s natural repair mechanisms, allowing for the insertion, deletion, or modification of genetic material. CRISPR-Cas9 enables precise genome changes, providing therapeutic solutions for genetic illnesses [98]. AI algorithms can design highly specific and efficient gRNAs by analyzing vast genomic datasets to predict the most effective sequences for targeting specific genes, minimizing off-target effects [99]. AI models trained to anticipate the potential off-target effects of CRISPR edits help researchers identify and mitigate unintended genetic modifications, improving the safety and precision of gene editing [100]. AI-driven tools are also being developed to optimize CRISPR components, such as Cas proteins and gRNAs, for better performance, enhancing the targeting range, editing efficiency, and specificity. AI can simulate the outcomes of CRISPR-based edits before conducting actual lab experiments, allowing researchers to test various strategies computationally and saving time and resources [101,102,103].

#### 3.2.4. Novel Computational Methods and Bioinformatics

The use of AI/ML in genomic data analysis has significantly enhanced our ability to analyze and interpret large datasets. ML algorithms can identify patterns and connections in genetic data that may not be apparent to traditional statistical methods, enabling the discovery of new biomarkers, therapeutic targets, and disease pathways [104]. The explosion of genomic data has necessitated the development of advanced big data analytics tools and platforms. Cloud computing and high-performance computing (HPC) infrastructures are increasingly used to store, manage, and analyze large-scale genomic datasets, facilitating collaborative research and integrating multi-omics data to enhance the understanding of complex biological systems [105]. Typical bioinformatics tasks in precision medicine include implementing and executing established and reproducible pipelines for analyzing genomic, transcriptomic, epigenomic, and proteomic data, as well as developing novel algorithms and tools for integrating and interpreting multi-omics data within a clinical context [106]. Additionally, developing robust bioinformatics pipelines and software tools is essential for the accurate and efficient analysis of genomic data. Widely used tools such as the Genome Analysis Toolkit (GATK), Burrows–Wheeler Aligner (BWA), and SAMtools are pivotal for sequence alignment, variant calling, and data processing. These pipelines are continually updated to incorporate new algorithms and improve performance [107].

## 4. Role of LLMs in Precision Medicine

LLMs are becoming increasingly crucial in genomic data analysis, enabling advanced tasks such as genetic variant annotation, gene expression prediction, and modeling gene-regulatory networks [108,109]. These models are trained on the extensive, diverse medical literature and datasets to utilize their advanced processing capabilities. This training helps them better analyze and generate written content, resulting in more accurate and efficient genomic interpretations. By incorporating attention mechanisms, LLMs can gain a nuanced understanding and generate relevant output, thereby significantly improving personalized medicine and genetic research.

### 4.1. Genomic Data Integration and Interpretation

Genomic data integration, which combines multiple sources such as DNA sequences, RNA transcripts, and epigenetic modifications, is crucial for comprehending biological systems fully [110]. GROVER, a foundation model with an optimized vocabulary for the human genome, was selected using next-k-mer prediction. This fine-tuning task is independent of the foundation model’s structure and can handle different vocabulary sizes and tokenization strategies without requiring the selection of models for specific biological tasks.

GROVER understands the DNA language structure by learning the characteristics of tokens and their sequence contexts [65]. Extracting this knowledge can create a grammar book for the code of life. This integrated approach helps extract critical insights for advancing personalized medicine. LLMs are capable of superior pattern recognition and contextual capabilities. DeepMAPs [111], a graph transformer-based method designed for integrating and interpreting biological networks from scMulti-omics data (including scRNA-seq, scATAC-seq, and CITE-seq), utilizes a graph with nodes that represent genes and cells, enabling features from various modalities to be mapped to genes [112].

### 4.2. Drug Development and Personalized Therapeutics

LLMs significantly enhance drug development and personalized therapeutics by utilizing genomic data to identify potential drug targets and predict individual responses to drugs. This capability enhances the efficiency of the development of personalized medications, lowers the risk of adverse reactions, and improves therapeutic outcomes. AlphaFold transformed the prediction of critical protein structures used in drug targeting, thereby simplifying development procedures [113]. Since then, several LLMs have been trained to achieve high accuracy. ProteinGPT is a multimodal LLM designed for protein property prediction and structure understanding, integrating protein sequence and structure encoders with linear projection layers and an LLM to generate precise and contextually relevant responses. Trained on a diverse set of 132,092 annotated proteins. These proteins are selected to cover various biological functions, structures, and properties, ensuring the model can handle various protein-related queries and analyses and optimizing the instruction-tuning process with GPT-4o [114]. Recent studies have shown that LLMs effectively predict individual patient responses to cancer treatments, indicating significant progress in precision medicine and personalized healthcare. CancerGPT, a few-shot learning approach with approximately 124M parameters, can successfully predict drug pair synergy in rare cancer tissues with limited data, producing results comparable to the larger GPT-3 model [115].

### 4.3. Integration of Multi-Omics Data

Multi-omics is an integrative approach that combines data from multiple “omics” disciplines, including genomics, transcriptomics, proteomics, metabolomics, and epigenomics, to comprehensively understand biological systems [1]. By leveraging these diverse datasets, researchers can elucidate complex molecular interactions and identify disease-associated biomarkers, enhancing diagnostic accuracy and therapeutic development. Multi-omics can be critical in tailoring treatment strategies to an individual’s unique genetic and biochemical profile, enabling more precise and practical interventions with reduced adverse effects. Integrating multi-omics data is crucial for comprehending complex biological processes, but traditional methods often struggle due to the heterogeneity and size of these datasets. LLMs can integrate various types of omics data, enabling a comprehensive understanding of genetic and molecular interactions. This integration enhances comprehension of disease mechanisms and identifies potential targets for therapeutic intervention. LLMs excel at handling large amounts of data and identifying patterns across multiple data types. Single-cell RNA sequencing (scRNA-Seq) has significantly contributed to our comprehension of cellular diversity and function [116]. Integrating LLMs into these frameworks can enhance multi-omics data analysis, yielding additional insights into gene regulation, cellular differentiation, and disease mechanisms. The incorporation of multi-omics data significantly improves the feature set used to train ML algorithms, resulting in more accurate models of disease risk, progression, and treatment responses [117]. LLMs can use these multi-omic datasets to improve prediction accuracy and aid in discovering new biomarkers, thereby advancing the field of personalized healthcare.

DeepMAPS is a graph transformer-based method for integrating and inferring biological networks from multi-omics data, such as scRNA-seq, scATAC-seq, and CITE-seq. It creates a graph with nodes representing genes and cells and maps features from other modalities to genes. DeepMAPS learns local and global features to construct cell–cell and gene–gene relationships by using RNA velocity to infer cell–cell communication [111]. scMoFormer is another advanced method that converts gene expression to protein abundance and facilitates multi-omics predictions, such as protein abundance to gene expression, chromatin accessibility to gene expression, and vice versa. It uses graph transformers to make these predictions, which improves the integration and interpretation of complex multi-omics data [118].

## 5. Challenges, Limitations, and Future of LLMs in Precision Medicine

AI integration in healthcare significantly impacts this field and yields high accuracy and efficiency. With the improving accuracy of various LLMs, health management is gradually moving towards higher efficiency, potentially at a lower cost, in the near future [119]. LLMs are valuable in analyzing genomic data and bioinformatics, leveraging extensive datasets to detect patterns that surpass traditional methodologies. LLMs have demonstrated impressive effectiveness in identifying complex patterns in genomic data, as evidenced by their ability to analyze and comprehend DNA and RNA sequences, much like textual data. Subsequently, there has been substantial advancement in tasks such as predicting splice sites, transcription factor binding sites, and other regulatory elements in the genome [120]. LLMs’ flexibility enables them to be utilized in various genomic tasks without requiring specific modifications. This adaptability is especially useful in genomics, where standard data types and analysis requirements are used. Although these models offer several advantages, they must be acknowledged and addressed to enhance the accuracy and effectiveness of diagnosis and treatment options in precision medicine. The limitations and potential research directions to mitigate these shortcomings are as follows.

### 5.1. Data Sparsity and Complexity

Data sparsity is a common challenge in genomic data, especially in single-cell omics data like scRNA-seq. Many genes are not expressed in most cells, leading to sparse data [110,121]. This poses difficulties for LLM algorithms, which typically work better with dense and evenly distributed data. The sparsity and high dimensionality of genomic datasets make it challenging to train models without overfitting or losing essential details in the noise. Even with tools like scBERT that are designed to tackle these issues, the underlying sparsity and expression level variability still pose significant computational challenges [122]. The Mixture of Experts (MoE) model, designed for efficiency and effectiveness, can understand context even with limited data through several mechanisms. By leveraging the sparse activation of experts, a sub-model dedicated to sub-tasks, only the most relevant experts are engaged for a given input, thus optimizing the use of available data [123]. The model’s dynamic routing mechanism further ensures that inputs are directed to the most appropriate experts, enhancing adaptability and context understanding. Efficient parameter usage allows the MoE model to generalize effectively. MoEs can be significantly helpful when handling limited genetic, clinical, or biological data by focusing on specialized experts tailored to specific datasets, maximizing the insights gleaned from restricted information, and improving predictive accuracy in sparse data scenarios.

### 5.2. Interpretability and Model Transparency

This is a significant limitation in genomic studies where understanding the biological implications of predictions is crucial. For instance, predicting interactions and comprehending biological pathways and underlying mechanisms are essential in gene regulatory network inference. The opaque nature of DL models, such as LLMs, can obscure these insights, making it challenging for researchers to trust and validate the results without extensive external testing.

Explainable AI (XAI) can enhance transparency and trust in LLM-based clinical decision support systems and biomedical research tools. XAI “explains” the thought process of an LLM or AI model in general decisions, leading to better diagnostic accuracy and patient outcomes. XAI also mitigates biases, ensuring equitable treatment. XAI methods like Local Interpretable Model-agnostic Explanations (LIMEs) and Shapley Additive explanations (SHAPs) have been applied to interpret AI models’ clinical decisions when predicting cardiovascular diseases and oncology decisions, improving trust in AI prediction [124,125].

### 5.3. Computational Resources

The training and fine-tuning of LLMs require significant computational resources. Many researchers cannot access state-of-the-art models due to the need for powerful GPUs and infrastructure. This is particularly challenging in genomics, where datasets can be extremely large. Additionally, training LLMs on genomic data often requires a significant initial computational investment and ongoing retraining as new data become available, further adding to the resource burden.

Several strategies alongside the supporting literature that mitigate the high resource requirements can be leveraged in precision medicine. Model pruning reduces the number of parameters in the model by removing less important weights or neurons, thereby minimizing computational resources without significantly affecting performance. Quantization lowers the precision of the model’s weights and activations, typically from 32 bit to 8 bit, which reduces memory usage and computational demands. Knowledge distillation involves training a smaller, more efficient model to mimic the behavior of a larger model, resulting in faster computations and reduced resource consumption [126,127]. Efficient model architectures, such as the Transformer-XL or Efficient Transformers, are designed with minimal computational overhead as a key requirement [128,129]. These strategies collectively contribute to reducing the computational load and enhancing the scalability of LLMs.

### 5.4. Relevance and Generalization Accuracy

The inherent differences between text and genomic data present significant challenges for LLMs’ effective generalization of genomic data. Text data are linear and sequential, whereas genomic data are three-dimensional, highly interactive, and non-linear. NLP-oriented models employed by models such as DNABERT may not fully capture the complexities of chromosomal interactions, epigenetic modifications, and the impact of non-coding regions [130]. The three-dimensional organization of genomic data within the cell nucleus is crucial for understanding gene regulation and genome function. Additionally, genes do not act in isolation, as there are complex interactions between different regions of the genome, including chromatin interactions and regulatory elements. Epigenetic modifications add another layer of regulation that is not present in text data. Furthermore, a significant portion of the genome consists of non-coding regions with regulatory functions.

Possible solutions to these challenges include the integration of multi-omics data to provide a more comprehensive understanding of biological systems and the development of advanced computational models that incorporate knowledge of the genome, such as ReUseData for efficient data management and reuse [131,132]. Promoting data sharing and collaboration among researchers can help overcome data scarcity and improve model training’s [133] 3D structure and epigenetic landscape, and the enhancement of data management tools.

### 5.5. Privacy and Security

The analysis of private health data using LLMs presents significant privacy challenges due to the sensitivity of healthcare data, including personal medical histories, diagnoses, and treatment plans. Unauthorized access or data breaches can lead to severe privacy violations and the misuse of sensitive information. Additionally, the complexity of LLMs and their decision-making processes can result in a lack of transparency and trust [134]. Algorithmic bias is another concern, as biased datasets can produce inaccurate or unfair outcomes, particularly for underrepresented demographic groups, leading to disparities in patient care and health outcomes. Additionally, protected data from research centers can become vulnerable to unauthorized usage for training, leading to potential financial losses [135].

To mitigate these privacy challenges, several solutions can be implemented. Robust security protocols, including encryption and regular audits, are essential to prevent unauthorized access. Transparency and accountability in data usage should be prioritized, with clear policies provided to patients. Privacy-preserving techniques like data anonymization, federated learning, and differential privacy can protect patient confidentiality while allowing for meaningful analysis. Collaboration among healthcare institutions, regulators, and AI developers is crucial for establishing robust governance frameworks and ensuring compliance with regulatory standards such as HIPAA. By addressing these challenges and implementing these solutions, LLMs can be effectively integrated into healthcare for precision medicine.

## 6. Conclusions

AI integration in healthcare has led to a paradigm shift in medical research and clinical decision processes. LLMs have a promising future in personalized medicine, with several key advancements on the horizon. They can integrate diverse datasets, such as genomic, proteomic, and clinical data, to identify patterns and correlations essential for understanding complex diseases and developing personalized treatments. Predictive modeling by LLMs forecasts disease progression, treatment outcomes, and potential side effects, enabling tailored treatment plans.

In drug discovery, LLMs expedite the identification of drug targets, predict interactions, and optimize formulations, thereby accelerating the development of new therapies. Clinical decision support from LLMs offers evidence-based recommendations, synthesizes medical research, and aids healthcare professionals in making informed decisions. Furthermore, LLMs enhance patient engagement by providing personalized health information, answering queries, and improving treatment adherence through conversational interfaces. They also support research by processing and summarizing vast amounts of the medical literature, providing easy access to knowledge. While promising, LLMs face challenges such as data privacy concerns, model interpretability issues, and the risk of generating inaccurate information. Ensuring responsible use and continuous improvement is crucial for their successful integration into precision medicine. Integrating multi-omics data, such as genomics, proteomics, and metabolomics, will provide a comprehensive understanding of diseases, enhancing treatment precision. Continuous enhancements to model architectures, such as transformer models, will increase the accuracy of understanding complex genetic data. Real-time data processing capabilities will emerge, enabling rapid insights and recommendations in healthcare conditions. Interdisciplinary collaboration among computational scientists, geneticists, and healthcare professionals will be essential for building therapeutically applicable models.

Validating LLM outputs, particularly in the genomic analysis field, involves a symbiotic operation between complementary technologies. Primarily, using high-quality, domain-specific datasets provides the knowledge base required to understand complex genomic patterns and biological terminology. Structured knowledge aggregation by symbolic-neural hybrid techniques and from dependable databases such as Gene Ontology or the Kyoto Encyclopedia of Genes and Genomes (KEGG) database is critical to increase its factual dependability. Retrieval-Augmented Generation (RAG) also increases accuracy by allowing the model to fetch and leverage contextual information from the current genomic literature in real time at inference. Adapter-based and fine-tuning methods facilitate the general-purpose LLMs to be trained into specialized genomic applications. In contrast, prompt engineering guides the model into biologically meaningful and structurally well-formed outputs. Moreover, uncertainty estimation methods, including ensemble methods and Monte Carlo dropout, enable the quantification of the confidence in predictions. Post-processing with biomedical ontologies and rule-based validators enables terminological correctness and consistency. XAI methods, such as attention visualization, increase interpretability and transparency for gene–disease association tasks. Feedback loops and continuous learning algorithms allow the model to stay in line with the latest genomic evidence. At the same time, strict compliance with regulations and ethics standards ensures that outputs are reliable for clinical decision-making.

Addressing ethical and privacy concerns with robust data protection measures will safeguard patient information. LLMs will also enhance personalized treatment recommendations, suggesting tailored therapies and lifestyle changes based on individual genetic profiles. Efforts to make these technologies scalable and accessible will broaden their impact, ensuring that the benefits are available to diverse populations. These improvements will collectively enhance the precision, efficacy, and accessibility of personalized healthcare, significantly improving patient outcomes.

## Figures and Tables

**Table 1:** A summary of LLMs.

Model	Developer	Key Features	Applications	Reference
BERT	Google	- Bidirectional pre-training - Uses masked language modeling and next-sentence prediction	Text classification, named entity recognition, chatbots, language translation	[34]
GPT	OpenAI	- Unidirectional autoregressive model - Uses decoder-only architecture	Text generation, language modeling, chatbots, creative writing	[41]
GPT-3	OpenAI	- Uses a very large-scale model with 175 billion parameters - Includes reinforcement learning from human feedback along with multi-modal support	Text generation, code generation, language translation, text summarization, chatbots	[37]
GPT-4	OpenAI	- Uses a very large-scale model, greater than GPT-3 - Autoregressive language model	Text generation, code generation, language translation, text summarization, chatbots	[42]
Text-To-Text Transfer Transformer (T5)	Google Research	- Uses a text-to-text framework where all NLP tasks are treated as text-generation tasks	Text translation and summarization, chatbots, text classification	[40]
RoBERTa	Meta AI	- Uses a BERT model with longer training, more data, and dynamic masking	Text classification, named entity recognition, chatbots, sentiment analysis	[35]
XLNet	Google/Carnegie Mellon University	- Combines autoregressive and autoencoding approaches - Uses permutation-based training	Text classification, sentiment analysis, chatbots	[43]
A Lite BERT (ALBERT)	Google and Toyota Technological Institute	- Uses parameter reduction techniques to lower memory consumption and increase training speed	Text classification, natural language inference, chatbots	[44]
BART	Meta AI	- Combines bidirectional and autoregressive transformers - Designed for sequence-to-sequence tasks	Text generation and summarization, machine translation, chatbots	[39]
ERNIE (Enhanced Representation through Knowledge Integration)	Baidu	- BERT-based model using phrase-level masking - Integrates external knowledge graphs during pre-training	Text classification, chatbots, natural language understanding, language generation	[45]
Turing-NLG	Microsoft	- Autoregressive language model - Very-large-scale model with 17 billion parameters	Text generation, chatbots, text summarization, dialogue systems	[46]

**Table 2:** DL models and LLMs for biological research and clinical decision processes.

Model	Developer	Key Features	Applications	Reference
ChemBERTa	Industry-Academic Collaboration	Self-supervised learning on SMILES strings	Lead identification, drug optimization	[49]
AlphaFold	DeepMind	DL for 3D protein structure prediction	Protein structure prediction, function understanding	[51]
GenoML	GenoML	ML for automated variant analysis	Variant annotation and prioritization in genomics	[53]
ProteinBERT	Industry-Academic Collaboration	BERT-based pre-trained on about 106M proteins from UniRef90	Protein function prediction, protein–protein interaction, drug discovery	[54]
ProtBERT	Industry-Academic Collaboration	BERT applied to protein sequences	Protein classification, function prediction, interaction analysis	[55]
DNABERT	Northwestern/Brook University	Transformer models for DNA sequences	Genomic variant identification, gene function prediction	[56]
MedBERT	Stanford University	BERT-based model pre-trained on electronic health records	Patient diagnosis prediction, treatment recommendation, medical image analysis	[60]
BioBERT	Naver/Korea University	BERT model pre-trained on biomedical literature from PubMed and PMC	Biomedical text mining, named entity recognition, relation extraction, interactive systems	[57]
PubMedBERT	Microsoft Research	BERT-based, pre-trained specifically on PubMed abstracts and full-text articles	Biomedical text mining, information retrieval, named entity recognition, relationship extraction	[58]
ClinicalBERT	MIT	BERT-based, pre-trained on clinical notes from electronic health records	Clinical text mining, patient outcome prediction, medical information extraction	[59]
GenoTEX	Collaborative Genomics Group	Benchmarking and LLM integration for gene expression data	Evaluation and benchmarking of LLMs in gene expression data analysis	[47]
QuST-LLM	QuPath and Bioinformatics	Spatial transcriptomics enhanced by LLMs	Analysis and interpretation of spatial transcriptomics	[61]
SeqMate	RNA-Seq Analysis Initiative	Automated RNA sequencing analysis pipeline with LLM support	RNA sequencing data preparation and differential expression analysis	[62]
GENA-LM	AIRI	Foundational DNA language model	Long DNA sequence handling	[63]
Geneverse	T Liu et al.	Multimodal LLM	Genomics and proteomics research	[64]
GROVER	German Cancer Research Center	DNA language model	Human genome context learning	[65]

**Table 3:** Summary of databases containing training datasets for genomic analysis for personalized medicine used for training DL and LLMs.

Dataset Name	Description	Source/Website
1000 Genomes Project	A comprehensive resource of human genetic variation, supporting studies on genetic variation, health, and disease. It includes data from diverse populations worldwide.	https://www.internationalgenome.org/data/ (accessed on 20 April 2025)
ENCODE Project	Provides functional genomic data, including ChIP-seq, RNA-seq, and epigenomic data, to identify all functional elements in the human genome.	https://www.encodeproject.org/ (accessed on 20 April 2025)
Genotype-Tissue Expression (GTEx)	Offers data on gene expression and regulation across 54 tissue sites from nearly 1000 individuals, enabling studies on tissue-specific gene expression.	https://www.gtexportal.org/home (accessed on 20 April 2025)
The Cancer Genome Atlas (TCGA)	Contains genomic, epigenomic, transcriptomic, and proteomic data for over 20,000 primary cancer and matched normal samples across 33 cancer types.	https://www.cancer.gov/ccg/research/genome-sequencing/tcga (accessed on 20 April 2025)
Human Microbiome Project	Provides data on microbial communities in the human body, including metagenomic and 16S sequencing data.	https://www.hmpdacc.org/resources/data_browser.php (accessed on 20 April 2025)
UniProt	A comprehensive database of protein sequences and functional information, supporting studies in proteomics and genomics.	https://www.uniprot.org/ (accessed on 20 April 2025)
dbSNP	A database of single-nucleotide polymorphisms (SNPs) and other genetic variations, facilitating studies on genetic associations and population genetics.	https://www.ncbi.nlm.nih.gov/snp/ (accessed on 20 April 2025)
Gene Expression Omnibus (GEO) Database	A repository for gene expression and other functional genomics data, supporting MIAME-compliant submissions and analysis tools.	https://www.ncbi.nlm.nih.gov/geo/ (accessed on 20 April 2025)
Catalogue of Somatic Mutations in Cancer (COSMIC)	An expert-curated database of somatic mutations in cancer, including mutation distributions and effects.	https://cancer.sanger.ac.uk/cosmic (accessed on 20 April 2025)
ClinVar	Archives information about genomic variations and their relationships to human health, including disease associations and drug responses.	https://www.ncbi.nlm.nih.gov/clinvar/ (accessed on 20 April 2025)
PharmGKB	A pharmacogenomics knowledge base that links genetic variations to drug responses, aiding in personalized medicine.	https://www.pharmgkb.org/ (accessed on 20 April 2025)
UK Biobank	A large-scale biomedical database containing genetic, lifestyle, and health data from 500,000 participants, supporting research in personalized medicine.	https://www.ukbiobank.ac.uk/ (accessed on 20 April 2025)
Medical Information Mart for Intensive Care (MIMIC)	A critical care database with de-identified health data, including clinical notes, lab results, and prescriptions, for personalized healthcare research.	https://mimic.mit.edu/ (accessed on 20 April 2025)
